# Moral Distress, Health and Intention to Leave: Critical Care Nurses’ Perceptions During COVID-19 Pandemic

**DOI:** 10.1177/23779608231169218

**Published:** 2023-04-17

**Authors:** Maria Andersson, Angelica Fredholm, Anna Nordin, Åsa Engström

**Affiliations:** 1 Swedish Red Cross University College, Huddinge, Sweden; 2 Department of Health, Education and Technology, Division of Nursing and Medical Technology, Lulea University of Technology, Luleå, Sweden; 3 County Council Värmland, Karlstad, Sweden; 4 Department of Health Science, Faculty of Health, Science, and Technology, Karlstad University, Karlstad, Sweden

**Keywords:** Covid-19 pandemic, critical care nurses, health, intensive care, moral distress

## Abstract

**Introduction:**

Moral distress increases the risk that critical care nurses will lose the ability to provide quality nursing care.

**Aims:**

To describe person-related conditions and perceptions of moral distress, health and intention to leave among critical care nurses in intensive care units, and to examine the relationship between person-related conditions, moral distress, health and intention to leave.

**Method:**

Cross-sectional, with 220 critical care nurses in 15 Swedish ICUs, and data gathered via a self-reported questionnaire.

**Results:**

Highest moral distress scores were reported in *futile care* and *poor teamwork* and 21% reported entertaining an intention to leave. Self-reported health was lower than before the COVID-19 pandemic and 4.1% reported pronounced exhaustion disorder. Self-reported health, reduced capacity to tolerate demands under time pressure, emotional instability or irritability, physical weakness, or being more easily fatigued and with decreased well-being were factors that had a relationship with *futile care.* Sleeping problems and intention to leave had a relationship with *poor teamwork.*

**Conclusions:**

Different strategies are needed to reduce moral distress and the leadership is crucial for managing crises such as the COVID-19 pandemic.

## Background

During the COVID-19 pandemic, moral distress (MD) was widely reported among critical care nurses (CCNs) working in intensive care units (ICUs) (Andersson et al., 2022a; Donkers et al., 2021; Guttormson et al., 2022; Petrișor et al., 2021; Rodriguez-Ruiz et al., 2022) and signaled ethical difficulties in the ICU environment. CCNs’ experience of MD increased the risk that CCNs would withdraw from their patients’ bedside, lose the ability to provide quality nursing care, experience decreased levels of health and well-being, and consider leaving their place of employment altogether (Andersson et al., 2022a; Petrișor et al., 2021; Rodriguez-Ruiz et al., 2022). The shortage of CCNs, especially in highly specialized environments such as intensive care, is a global issue (Khan et al., 2019; Xu et al., 2023), and during the COVID-19 pandemic the CCN shortage became noticeable amongst healthcare organizations worldwide.

During the pandemic, CCNs’ MD experiences resulted from their exposure to potentially harmful situations, for example, suboptimal nursing care (Andersson et al., 2022a; Silverman et al., 2021), concerns about seeing patients die without relatives at the patient's bedside (Donkers et al., 2021), colleagues not following safety guidelines or acting unsafely (Kok et al., 2021) and working with new colleagues without intensive care competence (Andersson et al., 2022a; Kok et al., 2021; Silverman et al., 2021). They also experienced ineffective communication and collaboration in ICU-teams (Andersson et al., 2022a; Silverman et al., 2021), conflicts between scarce resources and equal distribution (Silverman et al., 2021), suboptimal care due to lack of financial support (Kok et al., 2021), scanty resources of time (Kok et al., 2021) and staff shortages (Donkers et al., 2021).

### Moral Distress

MD was first described in nurses as a process of pain or anguish occurring when nurses knew the ethically correct action, they should provide in their work but were prevented from giving it due to real or perceived limitations (Jameton, 1985), such as self-doubt, perceived powerlessness, inadequate staffing, lack of administrative support or hierarchies within the healthcare system (Hamric et al., 2012). These internal and external constraints prevented the nurses from acting correctly in accordance with their ethical values (Boyle & Bush, 2018; Jameton, 1985).

Since this first definition of MD by Jameton (1985), other authors have made contributions to refinement of the concept of MD by adopting broader definitions. Fourie (2015) argued that moral constraint should not be a necessary condition of MD, but that moral conflict should be included as a potential cause of distress. Morley et al. (2020) argued that Jameton's (1985) definition of MD might fail to capture CCNs’ experiences and suggested a broader definition of MD that included a variety of moral events, such as moral tension, moral uncertainty, moral constraint, moral conflict, and moral dilemmas (Morley et al., 2020).

Nonetheless, there is broad agreement that CCNs are at risk for MD (McAndrew et al., 2018), and the intensity of the experience of MD might increase to a point and then decrease as the acute phase of the MD situation passes—this is the crescendo of MD. The feelings and personal discord from the morally distressing situation continue after the situation is over and this residual distress acts as a new baseline from which the next crescendo of MD builds. However, according to McAndrew et al. (2018), it is unclear whether MD intensity increases during the time of work or if MD intensity decreases over time. No matter how it plays out over time, MD might have an impact on a nurse's health and well-being, especially when the nurse is repeatedly exposed to morally distressing situations (McCarthy & Gastmans, 2014).

### Health

A sampling of health professionals (*n*  =  34) described the concept of health as a multi-faceted concept emerging as a subjective assessment and saying that health is about one's whole life (Johansson et al., 2009). This description resonates well with WHO's (1948) definition of health: “Health is a state of complete physical, mental and social well-being and not merely the absence of disease or infirmity.” Health can be described as an experience of balance and being in equilibrium with those around you and with life in general (Dahlberg & Segesten, 2010). Petrișor et al. (2004) has described health from a holistic perspective as well-being and balance between a person's ability to act and to realize one's vital goals. CCNs in the frontline against COVID-19 were exposed to high levels of stressful and traumatic events (Selman et al., 2020), and studies (Crowe et al., 2021; Guttormson et al., 2022; Kok et al., 2021; Petrișor et al., 2021; Romero-García et al., 2022) have shown that CCNs’ MD is associated with negative psychological outcomes which might affect their health. The prevalence of anxiety, depression (Crowe et al., 2021; Guttormson et al., 2022; Petrișor et al., 2021), posttraumatic stress (Crowe et al., 2021; Guttormson et al., 2022), and burnout symptoms increased among CCNs (Guttormson et al., 2022; Kok et al., 2021). CCNs’ health and well-being are vital for allowing them to provide quality nursing care and to retain their employment in ICUs.

On an organizational level, a high turnover among CCNs might compromise the nursing care quality in ICUs and in turn aggravate CCNs experience of MD. According to Rhéaume et al. (2022), the COVID-19 pandemic has left many CCNs in deep distress. However, unwavering support from hospital administrators is required to address CCNs’ physical and mental difficulties—such as exhaustion and burnout—and promote the consistent provision of high-quality nursing care in ICUs. By identifying MD situations that have distressed CCNs and affected their health, it will be possible to suggest interventions targeting mitigation of their negative consequences by providing a safe care environment and improving their well-being. A lack of knowledge about MD and health among CCNs working in Swedish ICUs during the COVID-19 pandemic and the relationships between MD, health and intention is, however, hindering such interventions.

The aim of this study was to describe person-related conditions and perceptions of MD, health, and intention to leave among CCNs in ICUs, and to examine the relationship between person-related conditions, MD, health and intention to leave.

## Method

The study was cross-sectional and a part of a larger study, whose aim was to explore challenges for CCNs during the COVID-19 pandemic.

### Setting

ICUs with more than 40 care sessions related to patients with COVID-19 diagnosis were identified by the Swedish Intensive Care Registry. The identified ICUs represented university hospitals (teaching hospitals that provide highly specialized care), county hospitals (that provide general and, to some extent, specialized care), and local hospitals (that provide general care). In Swedish ICUs, the nurse-to-patient ratio is normally 1:1–2 and the ICU team caring for critically ill patients consists of CCNs with postgraduate education in intensive care, enrolled nurses, specialist physicians, and physiotherapists. During the COVID-19 pandemic, several ICUs in Sweden temporarily needed to change the competence mix in the ICU team and included registered nurses with another postgraduate education as well as those without any postgraduate education at all.

### Sample

The sample consisted of consecutively invited participants from 15 ICUs in different counties in middle and northern Sweden and focused on CCNs who were working in ICUs during the second year of the COVID-19 pandemic.

### Instruments

Participants’ person-related conditions comprised age, gender, household, postgraduate education in intensive care, years of experience in ICUs, increased responsibility for nursing care, introduction of new co-workers, and supervision of intensive care students (see Table 1).

**Table 1.** CCNs’ (*n*  =  220) Person-Related Conditions.

Variable	Total *n* (%)	M (SD)	Range (min–max)
Age		46.50 (10.76)	28–65
Gender			
Women	186 (84.5)		
Men	32 (14.5)		
Household			
Single without children	24 (10.9)		
Single with children	21 (9.5)		
Cohabiting without children	60 (27.3)		
Cohabiting with children	114 (51.8)		
Post-graduate education in intensive care			
Yes	217 (98.6)		
No	3 (1.4)		
Years of experience in ICU		13.61 (10.12)	0–45
Increased responsibility for nursing care			
Yes	171 (77.7)		
No	49 (22.3)		
Introducing new co-workers to the ICU			
Yes	192 (87.3)		
No	28 (12.7)		
Supervised intensive care nursing students			
Yes	103 (46.8)		
No	117 (53.2)		

MD was measured with the Italian version of the Moral Distress Scale–Revised (MDS-R) (Lamiani et al., 2017). The MDS-R has been identified as one of the most useful and appropriate instruments for research purposes (Giannetta et al., 2020; Lamiani et al., 2017).

The MDS-R consists of 14 items, related to the four MDS-R dimensions: *futile care* (three items), *ethical misconduct* (five items), *deceptive communication* (three items), and *poor teamwork* (three items). The frequency (i.e., how often the situation arose) and the level of intensity (i.e., how disturbing the situation was when it occurred) of each item was evaluated using a five-point Likert scale; frequency ranged from 0 (never) to 4 (very frequently) and the level of disturbance ranged from 0 (none) to 4 (to a great extent) (see Table 2).

**Table 2.** Description of CCNs’ Perceptions of Moral Distress.

Dimensions with items	Range	Mean (SD)	Rank
*Dimension: Futile care*		5.42(3.38)	
2. Follow the family's wishes to continue life support, even though I believe it is not in the best interest of the patient	0–16	5.07(3.77)	3
3. Initiate extensive life-saving actions when I think they will only prolong death	0–16	5.75(4.22)	1
6. Continue to participate in the care of a hopelessly ill person who is being sustained on a ventilator when no one will make a decision to withdraw support	0–16	5.32(4.11)	2
*Dimension: Ethical misconduct*		**-**	
5. Feel pressure from others to order what I consider to be unnecessary tests and treatments	0–16	3.11(3.35)	7
7. Avoid taking action when I learn that a physician or nursing colleague made a medical error and did not report it	0–16	1.78(2.36)	9
9. Increase the dose of sedatives/opiates for an unconscious patient when I believe doing so could hasten the patient's death	0–16	0.98(1.87)	13
10. Take no action on an observed ethical issue because the involved staff member or someone in a position of authority requested that I do nothing	0–9	0.55(1.40)	14
11. Follow the family's wishes for the patient's care when I do not agree with them because of the fear of a lawsuit	0–16	1.08(2.27)	12
*Dimension: Deceptive communication*		**-**	
1. Witness healthcare providers giving ‘false hope’ to the patient or family	0–12	2.18(2.62)	8
4. Follow the family's request not to discuss death with a dying patient when they ask about dying	0–16	1.50(2.48)	10
14. Ignore situations in which patients were not given adequate information to ensure informed consent	0–12	1.17(1.95)	11
*Dimension: Poor teamwork*		4.16(3.18*)*	
8. Assist another physician or nurse who, in my opinion, is providing incompetent care	0–16	4.53(3.89)	4
12. Watch patient-care quality suffer because of a lack of provider continuity	0–16	4.28(3.86)	5
13. Witness diminished patient-care quality due to poor team communication	0–16	3.71(3.66)	6
Total score	2–135	41.73(23.46)	

Score range 0–16 (higher scores reflect more moral distress).

The Italian version of the MDS-R was translated into Swedish according to Brislin's (1970) translation model. The MDS-R was translated from Italian to Swedish by a bilingual translator with Italian as their native language, while the back translation, from Swedish to Italian, was done by the translator and the members of the research team. To test face and content validity, a pilot test was conducted and tested on four CCNs who were asked to judge whether the items were understandable and clear. This pilot resulted in minor linguistic changes and layout changes.

The MD score of each item resulted from multiplying the frequency of occurrence and level of intensity (range 0–16), which was then summed up to yield a total score range from 0 to 224 with 0 indicating no perceived MD and 224 indicating the highest level of MD. The mean value of the scores for each item and dimension was calculated by adding the item scores and dividing by the number of items answered.

The original reliability in the psychometric testing of the Italian version of MDS-R yielded a Cronbach's alpha of 0.81 (Lamiani et al., 2017). In this study, MDS-R reliability was supported with a Cronbach's alpha of 0.81, and in each dimension *futile care* of 0.78, *ethical misconduct* of 0.49, *deceptive communication* of 0.37, and *poor teamwork* of 0.78. Since the Cronbach's alpha values were less than 0.70 in the MDS-R dimensions *ethical misconduct* and *deceptive communication*, statistical analysis was performed only at item-level.

Intention to leave was measured by two questions. The question “Have you ever considered leaving your position because of moral distress?” could be answered in three ways: “Yes, I have left a position before”; Yes, I have considered it before”; or “No, I have never considered leaving.” The question “Are you currently considering leaving your current position in the ICU because of MD?” could be answered in two ways—“Yes” or “no”.

Participants self-reported health right now and before the COVID-19 pandemic was measured with two questions. The responses were measured using a five-point Likert scale ranging from 1 (poor) to 5 (excellent). Health was also measured with the ISM-instrument Self-rated Exhaustion Disorder (s-UMS) (Institute for Stress Medicine, Gothenburg/ Göteborg, 2016).

The s-UMS consists of four questions about different symptoms of exhaustion disorder. Three questions with six sub-questions can be answered in two ways (“yes” or “no”), and one question can be answered in three ways: “Yes, to a great extent”; “Yes, somewhat”; or “No, not at all” (see Table 3).

**Table 3.** Description of CCNs Self-Rated Exhaustion Disorder.

Questions	Total *n* (%)	*p*-value
1. Do you currently feel, and have felt for more than 2 weeks, physically and/or mentally exhausted?		
- No	82 (37)	
- Yes	137 (63)	<.001
2. Do you consider this exhaustion to be caused by long-term stress exposure (that you have been exposed to great strain or experienced pressure for 6 months or more		
- No	53 (28)	
- Yes	133 (72)	<.001
3. During the last 2 weeks, have you experienced:		
a. Concentration or memory problems?		
- Yes	95 (44)	
- No	123 (56)	.058
b. Markedly reduced capacity to tolerate demands or to work under time pressure?		
- Yes	72 (33)	
- No	146 (67)	<.001
c. Emotional instability or irritability?		
- Yes	130 (60)	
- No	88 (40)	<.004
d. Sleeping problems?		
- Yes	95 (43)	
- No	124 (57)	.050
e. Physical weakness or being more easily fatigued?		
- Yes	116 (53)	
- No	102 (47)	.343
f. Physical symptoms such as muscular pain, chest pain, palpations, gastrointestinal problems, vertigo, or increased sensitivity to sounds?		
- Yes	110 (50.5)	
- No	108 (49.5)	.892
4. Have the complaints above (questions 1–3) markedly decreased your well-being and/or your functional capacity (workability, family life, leisure activities or other important ways)?		
- Yes, somewhat or to a great extent	148 (68.8)	
- No, not at all	67 (31.2)	<.001
Exhaustion disorder syndrome		
- Mild/moderate exhaustion	8 (3.6)	
- Pronounced exhaustion	9 (4.1)	

Statistical analysis—Pearson Chi-square test. Statistical significance at *p* < .05.

Participants who answered “Yes” to the first two questions and “Yes” in at least four of the six sub-questions in question 3 meet the criteria for self-reported exhaustion disorder syndrome, if they also answered “Yes, somewhat” or “Yes, to a great extent” in question 4. Question 4 discriminates between mild and moderate exhaustion disorder and pronounced exhaustion disorder (Institute for Stress Medicine, Gothenburg/Göteborg, 2016).

### Data Collection

Permission to conduct the study was obtained from the heads of the ICU departments. Each ICU head appointed one CCN who acted as a liaison between the researchers and the ICUs. The appointed CCN gave information verbally and in writing to the participants and gave information about the study through ICU newsletters and staff rooms. A questionnaire package with an addressed and prepaid envelope was placed in each participant's post office box in the ICU. Along with the questionnaire package, the participants received written information about the study. Two reminders were sent via e-mail to the appointed CCN who put up reminders on the ICU notice board. The participants returned the completed questionnaires to one of the researchers. Due to the heavy workload in Swedish ICUs, data were collected over a broad period from July 2021 to November 2021.

### Data Analysis

CCNs’ personal-related conditions and perceptions of MD, health and intention to leave, were examined with descriptive statistics. Pearson's chi-square and student's *t*-test were used to examine differences in health. The relationships between the MDS-R dimensions *futile care* and *poor teamwork,* single items in the dimensions *ethical misconduct* and *deceptive communication*, person-related conditions, health and intention to leave were examined with linear regression. Cronbach's alpha was used to test reliability, and statistical significance was set at *p* < .05. IBM SPSS version 28 (IBM, Armonk, NY, USA) was used for all analyses.

### Ethical Considerations

Ethical approval for the study to be conducted was granted by the Ethics Review Authority (reg. no. 2020-04428). The heads of each ICU department gave their permission to conduct the study. Participation in the study was voluntary, the participants’ identities were kept confidential and informed consent was indicated by returning the questionnaire.

### Results

Two hundred and twenty out of 733 CCNs participated in the study (response rate 30%). They were 28 to 65 years of age, and 84.5% were women. Approximately half of the CCNs were cohabiting with children and 99% had education in intensive care on an advanced level. CCNs had on average 13.6 years of ICU experience and most participants (78%) reported an increased nursing care responsibility. A majority (87.3%) of CCNs had introduced new co-workers and 46.8% had supervised postgraduate critical care nursing students (see Table 1).

### Moral Distress

The mean scores for MDS-R's dimensions *futile care* were 5.42 (SD 3.38) and for *poor teamwork* 4.16 (SD 3.18). For the five items in the dimension *ethical misconduct*, the highest mean score (3.11 SD 3.35) was the item related to “feel pressure from other…” and the other four items’ mean score was <2. For the three items in the dimension *deceptive communication*, the highest mean score (2.18) was the item related to “witness healthcare providers giving false hope…,” and the other two items’ mean score was <2 (see Table 2).

CCNs rated the item “initiative extensive life-saving actions when I think they will only prolong death” highest and the item “take no action on an observed ethical issue…” as lowest (see Table 2).

From the 216 CCNs, 119 (55%) of them reported past or present thoughts about leaving their job. From these, 28.5% had considered leaving a position in the past because of MD, 5.6% had left a position before because of MD and 21.3% were considering leaving their current position because of MD. Thus, a total number of 170 CCNs (78.7%) had no intention to leave their current position in the ICU because of MD.

### Health

The mean value for CCNs’ self-reported health right now (mean 3.15, SD 0.87) was significantly lower (*p*-value < .001) than before the COVID-19 pandemic (mean 3.97, SD 0.73). Statistically significant differences existed among CCNs when comparing self-rated exhaustion disorder. A majority (63%) of CCNs had felt physically and psychologically exhausted for more than 2 weeks and 72% perceived this exhaustion as being due to prolonged stress. Sixty percent experienced more emotional instability or irritability, but most CCNs did not experience any reduced capacity tolerating work under time pressure (67%), nor sleeping problems (57%). A total of nine out of 220 CCNs (4.1%) reported pronounced exhaustion disorder (see Table 3).

### Relationships

Table 4 presents regression coefficients and significant differences comparing CCNs’ perceptions of MD for the MDS-R dimensions *futile care* and *poor teamwork.* The six highest rated MDS-R items were related to the dimensions *futile care* and *poor teamwork* and therefore only regressions were made with these two dimensions. The analysis was controlled for differences across CCNs’ person-related conditions.

**Table 4.** Relationships Between Symptoms of Exhaustion Disorder, Health, Intention to Leave a Current Position now or in the Past and CCNs Perceptions of Moral Distress in the MDS-R Dimensions Futile Care and Poor Teamwork.

Independent variable	Dependent variable	Unstandardized β	*p*-value
Reduced capacity to tolerate demands or to work under pressures	Futile care	1.852	.002
Emotional instability or irritability	Futile care	1.932	<.001
Physical weakness or being more easily fatigued	Futile care	1.264	.01
Decreased well-being or functional capacity	Futile care	1.946	<.001
Sleeping problems	Poor teamwork	.972	.046
Health right now	Futile care	−1.146	<.001
Intention to leave current position	Poor teamwork	1.214	.025

Statistical analysis—linear regression. Controlled for following person-related conditions: age, gender, household, post-graduate education in intensive care, years of experience in ICU, increased responsibility for nursing care, orientation new co-workers and supervision intensive care students. Health right now, self-rated exhaustion disorder and intention to leave with significance at *p* < .05 are listed.

CCNs with higher self-reported health rated MD lower in the MDS-R dimension *futile care* than those with lower self-reported health. CCNs who self-rated reduced capacity to tolerate demands under time pressure, emotional instability or irritability, physical weakness, or being more easily fatigued and with decreased well-being rated MD higher in the MDS-R dimension *futile care* than those who had self-rated otherwise. CCNs who self-rated sleeping problems and intended to leave their current position in the ICU, rated MD higher in the MDS-R dimension *poor teamwork* than those who reported no sleeping problems and had no intention of leaving their current position.

## Discussion

### Moral Distress

CCNs’ highest ranking MD situations were related to MDS-R dimensions *futile care* and *poor teamwork,* and this is in line with previous studies (Petrișor et al., 2021; Rodriguez-Ruiz et al., 2022). However, in our study, the patient-related factors seem to be the most important root factors inducing the highest MD, in contrast to Petrișor et al. (2021) and Rodriguez-Ruiz et al. (2022), who reported higher MD related to system-associated factors.

It is important to be aware that the studies of Petrișor et al. (2021) and Rodriguez-Ruiz et al. (2022) report CCNs’ MD perceptions during the first pandemic year, while our study gathered data from the second pandemic year. This difference might reflect that the new and rapidly changed circumstances that the healthcare system and specifically the ICUs faced (Andersson et al., 2022b) influenced the Swedish CCNs less during the second year, while they were more affected by patient-related factors, such as not being able to offer person-centered care (Andersson et al., 2022a). It seems that the crescendo of MD not just increased over time, but rather it also changed important root factors over time.

CCNs experienced MD related to *poor teamwork* and decreased care quality. Uncertainty about team members’ knowledge and skills in intensive care (Donkers et al., 2021; Petrișor et al., 2021; Rodriguez-Ruiz et al., 2022; Silverman et al., 2021) highlights the importance of communication within the ICU team during the pandemic (Andersson et al., 2022a; Bergman et al., 2021).

According to Ervin et al. (2018), ICU teams are distinguished from other healthcare teams in that they are low in temporal stability, which can impede important team dynamics. ICU teams must work in physically and emotionally challenging environments. The importance of sharing information and the decision-making processes is obvious, and there are potential barriers to successful team performance, including the lack of effective conflict management and the presence of multiple goals, which are sometimes conflicting.

Our study shows that CCNs can experience MD on the individual, unit and organizational levels, and according to Cronqvist et al. (2004), MD neither can nor should be totally illuminated in intensive care. However, MD needs to be handled in such a way that it will be beneficial for the individual as well as the organization (Cronqvist et al., 2004). It is important that healthcare managers ensure time for CCNs to reflect on their experiences, and, according to Greenberg (2004), reflection is necessary to be able to create a meaningful rather than traumatic narrative.

### Intention to Leave Current Position

Sleeping problems and MD related to *poor teamwork* was associated with an intention to leave the current position. According to Petrișor et al. (2021), MD and its included system-related factors distinguish between CCNs that demonstrate depression and/or anxiety symptoms, while system-related factors differentiate those intending to leave the current job from those who do not. Crowe et al. (2022) conclude that the mental health toll of the pandemic has been significant for Canadian CCNs and highlights the need for personal aid and system-level changes. Qualitative analysis of written comments described an immense mental health toll on CCNs that stemmed from unsuccessful leadership and the stressful nature of the work environment, which led to a sense of disappointment and an intention to leave (Crowe et al., 2022).

No manager was prepared for the COVID-19 pandemic and most important qualities of a leader in times of crisis are, according to Hayes and Cocchi (2022), presence, transparency, and empathy. Previous studies (Andersson et al., 2022b; Guttormson et al., 2022) have indicated a sometimes-invisible leadership. Rhéaume et al. (2022) state that healthcare organization leaders must be present to answer questions, listen to staff, and be able to discuss ethical issues. The responsibility of nurse managers as patient–nursing staff facilitators need to be emphasized to provide best possible response in a crisis. Vázquez-Calatayud et al. (2022) has suggested development of training strategies for nurse managers in emotional self-management and advertising a visionary approach.

However, a Swedish study by Gadolin et al. (2022) has shown that nurse managers emphasized the importance of being available to their staff. They tried to achieve this and compensated for insufficient availability. On the other hand, being highly available, providing daily support and meeting individual as well as collective needs might be overwhelming for nurse managers (Gadolin et al., 2022). There is a need for further investigation about ICU nurse managers and their experiences during the COVID-19 pandemic to be better prepared in the future when crises arise.

### Health

In our study, the CCNs self-reported their general health had been higher before the COVID-19 pandemic than during the second year of it and that they had different symptoms of exhaustion. A few CCNs (4.1%) had pronounced exhaustion disorder with an increased risk for sick leave. The most prevalent risk factors for stress-related health concerns are high workload, emotional demands (Hasselberg et al., 2014), lack of support, lack of work control (Aronsson et al., 2017), and an imbalance between work and recovery (Håkansson & Ahlborg, 2017). A CCN's exhaustion symptoms might also indicate compassion fatigue, a consequence according to Coetzee and Klopper (2010) of stress felt by nurses and a function of their intense engagement with patients over time with limited possibilities for rest and recovery.

Compassion is often defined as a “moral emotion” required for nurses to provide nursing care with quality (von Dietze & Orb, 2000) and is seen by nurses as an empathic gift (Gustafsson and Hemberg, 2022). Shin and Yoo (2022) have described nurses during the COVID-19 pandemic who constantly provided nursing care with a sense of calling and responsibility, and Andersson et al. (2022b) described a CCN who said, “Just a lot of oxygen and abdominal positions and routines and so on…but being good at intensive care nursing is so much more than that.” Ruiz-Fernández et al. (2020a) showed that health professionals working in specific COVID-19 units and in emergency wards had increased risk for compassion fatigue and burnout.

Compassion fatigue, or secondary traumatic stress, is the cost of caring for others or their emotional pain, resulting from the desire to relieve the suffering of others (Ruiz-Fernández et al., 2020b). It gives rise to emotions (e.g., guilt, shame, sadness, irritation, and impatience) in the nurse, both as a professional and as a person (Gustafsson & Hemberg, 2022). CCNs in the present study reported physical as well as psychological exhaustion that also might indicate compassion fatigue. Important aspects for nurses not to end up in chronic compassion fatigue are a healthy lifestyle, time for reflection, recovery and social networking (Gustafsson & Hemberg, 2022). Health professionals’ health and well-being improve their work (Galletta et al., 2016), and it is a nursing management responsibility to create a care environment in which CCNs can work without negative psychological outcomes affecting their health.

## Strengths and Limitations

CCNs from different ICUs located in various counties in middle and northern Sweden participated in the study. As with many institution-wide surveys, the response rate was low and might represent a selection bias. This suggests a dropout analysis, but such an analysis is not possible, owing to incomplete data. It should be noted that the selected person-related conditions did not cover all aspects of work, missing out, for example, on culture, character, and economics. The study provided no explanation of the specific ICUs’ working environment, such as how nursing care was organized, how many beds were in each unit and the average length of a patient's stay in each ICU.

The Italian version of MDS-R has not been used in Swedish ICUs previously or for CCNs only and needs to be further psychometrically tested. S-UMS is an assessment instrument based on the exhaustion disorder criteria and assists physicians in the diagnosis of exhaustion disorder. Other instruments can be used for the same assessment, for example, the burnout assessment tool (Schaufeli et al., 2020), a validity- and reliability-tested instrument that is available in several languages.

## Conclusions

This study shows an existing relationship between health and MD, both in terms of general health and of self-rated exhaustion disorder. CCNs who self-rated sleeping problems and had an intention to leave their current position in an ICU, rated MD higher relating to poor teamwork than those who reported no sleeping problems and had no intention to leave their current position. Patient-related factors induced a higher degree of MD than system-related factors, possibly indicating the need for CCNs to be able to provide person-centered nursing care.

Leadership is crucial for managing crises such as the COVID-19 pandemic. Plans are needed about how to best support both the patients and the nurses in such dire times. Reduced MD improves retention and sustainability of the workforce, meanwhile providing benefits for the patients as well as for the CCNs. Powerful nurses’ representation in policy decision-making is necessary for nurse involvement and to give a voice to the nursing care. This will help to develop preparedness for future crises and to value the past experiences of nurses helping to build a system that acknowledges vulnerabilities exposed during the COVID-19 pandemic. Nurse managers and leaders should consider these findings when developing strategies to improve CCNs’ health and well-being.

## Implications for Practice

- Impaired health and well-being express itself in various ways and not only in symptoms but also in emotions that might be just as devastating for CCNs’ professional role and their intention to leave their current job.
- The possibility to provide person-centered nursing care and possibility to influence the patients’ care will probably reduce the CCNs sense of MD, and by so doing decrease the risk of their intention to leave.
- Reduced MD improves sustainability of the personnel and provides benefits for the patients as well as for the CCNs.
- Nurses’ representation in policy decisions are necessary for nurse involvement and to give voice to the nursing care.
- Great leadership and resource management are vital, especially during crisis such as a pandemic.
